# Supplementary material for: Adducin‐1 is essential for spindle pole integrity through its interaction with TPX2
Source: EMBO Rep. 2018 Jun 19;19(8):e45607. doi: 10.15252/embr.201745607 (PMC6073210; doi:10.15252/embr.201745607)
Supplement: Supplementary file 1 — Appendix [file EMBR-19-e45607-s001.pdf]

Appendix for

Adducin-1 is essential for spindle pole integrity through its interaction with TPX2

Wen-Hsin Hsu, Won-Jing Wang, Wan-Yi Lin, Yu-Min Huang, Chien-Chen Lai, Jung-Chi Liao, and Hong-Chen Chen*

*Corresponding author. Email: hcchen1029@ym.edu.tw

This PDF file includes:

Appendix Figure S1

Appendix Figure S2

Appendix Figure S3

Appendix Figure S4

Appendix Figure S5

A

residue	phosphopeptide
S12	AAVVTpSPPPTTAPHK
S358	SPGpSPVGEGTGSPPK
S355/S358	SRpSPGpSPVGEGTGSPPK
S465	GDEASEEGQNGSpSPK
S726	TPpSFLKK

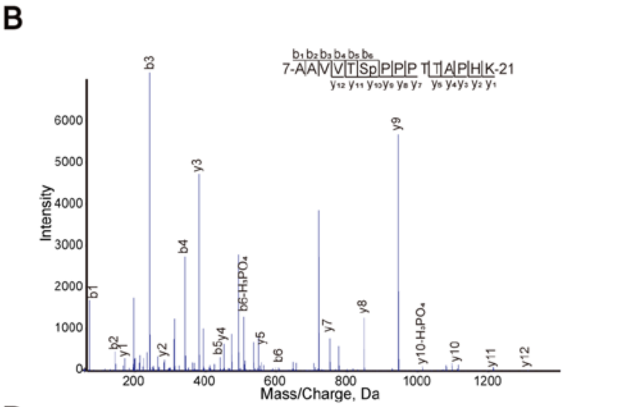

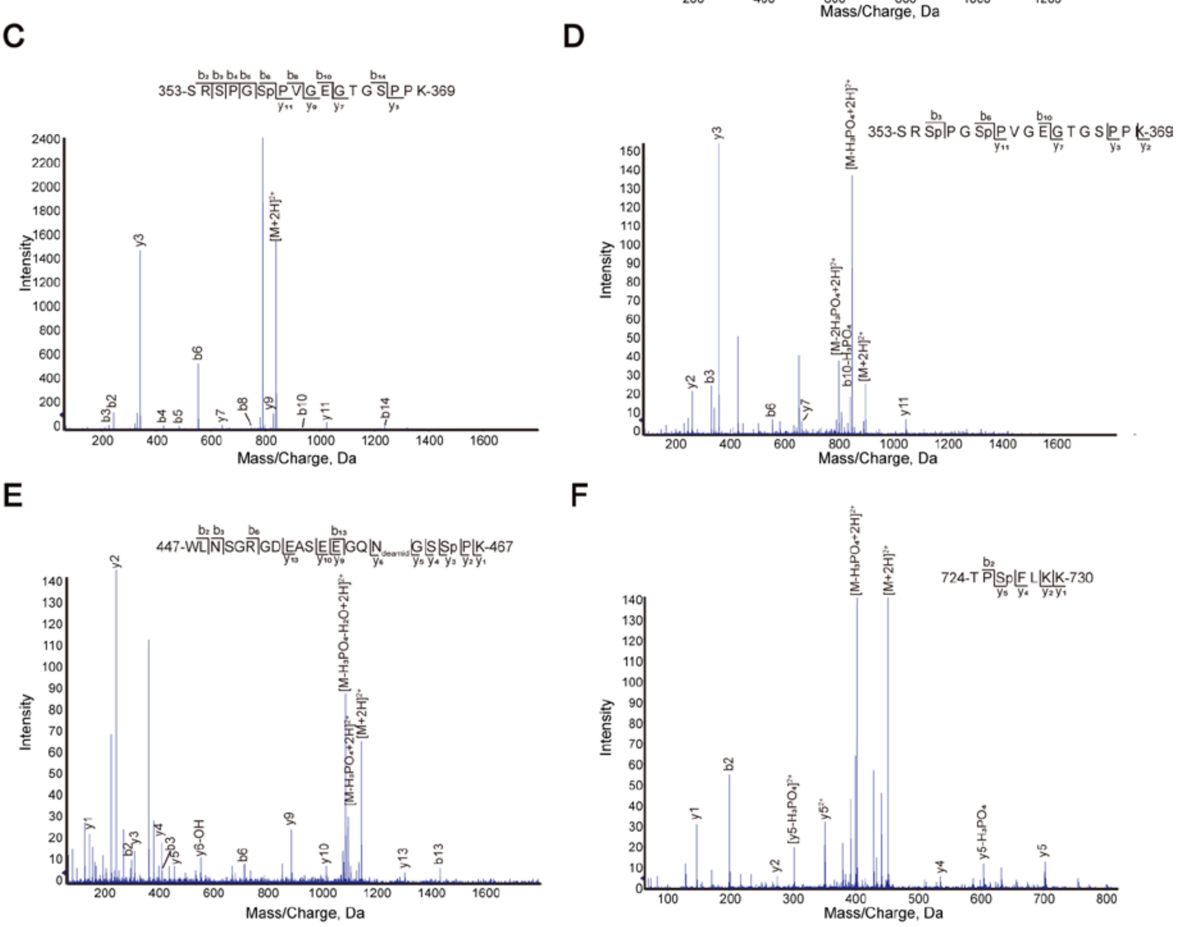

Appendix Figure S1. Identification of ADD1 phosphorylation sites in mitotic HeLa cells by mass spectrometry. (**A**) Summary of the phosphorylation sites identified by mass spectrometry analysis. (**B-F**) The mass spectrum of the identified phospho-peptides derived from ADD1.

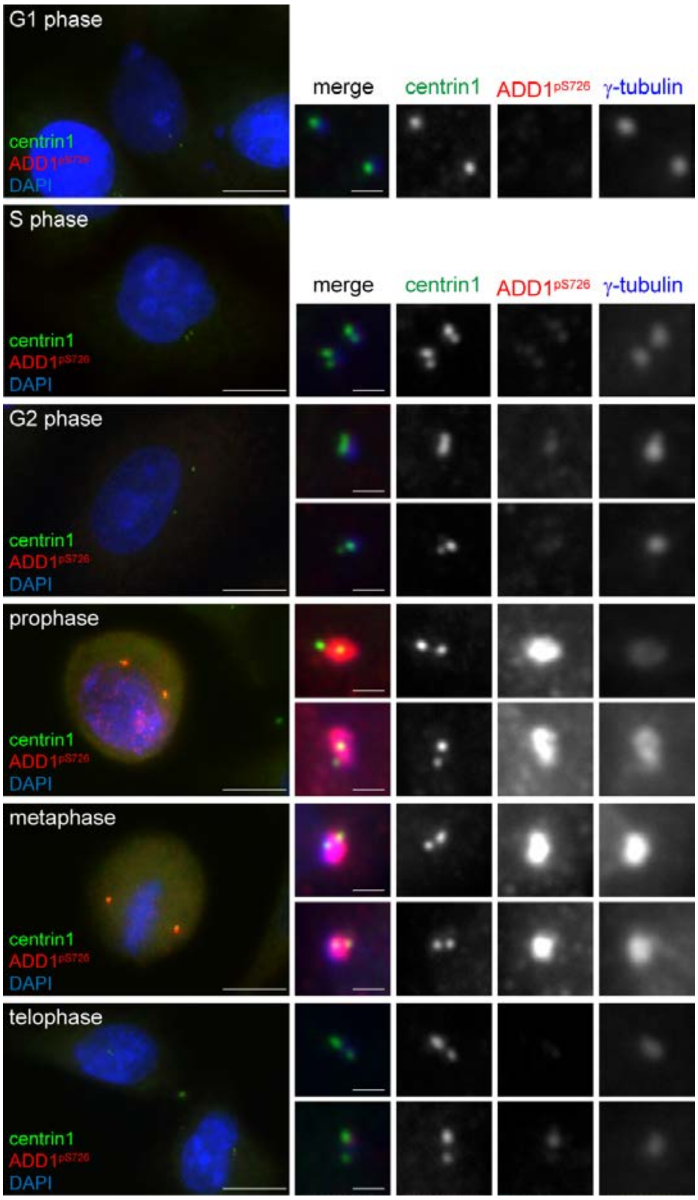

Appendix Figure S2. ADD1 S726 phosphorylation is increased during mitosis in HeLa cells. HeLa cells at the indicated cell-cycle phases were stained for ADD1 pS726, centrin1, γ-tubulin, and DNA. High magnification images of each centrosome are shown. Centrin1 is a marker for centrioles. Scale bars, 10 μm (main image) and 1 μm (zoomed images).

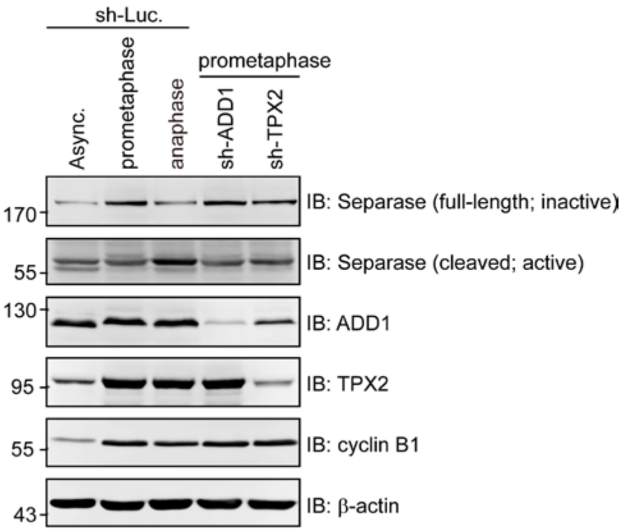

Appendix Figure S3. ADD1 or TPX2 depletion does not induce premature separase activation. HeLa cells were infected with lentiviruses expressing shRNAs to ADD1 (sh-ADD1), TPX2 (sh-TPX2), or luciferase (sh-Luc.). The cells remained asynchronized (Async.) or were synchronized at prometaphase by nocodazole. The prometaphase-arrested cells proceeded to the anaphase after nocodazole was removed for 2 h. Equal amounts of whole cell lysates were analyzed by immunoblotting with the indicated antibodies.

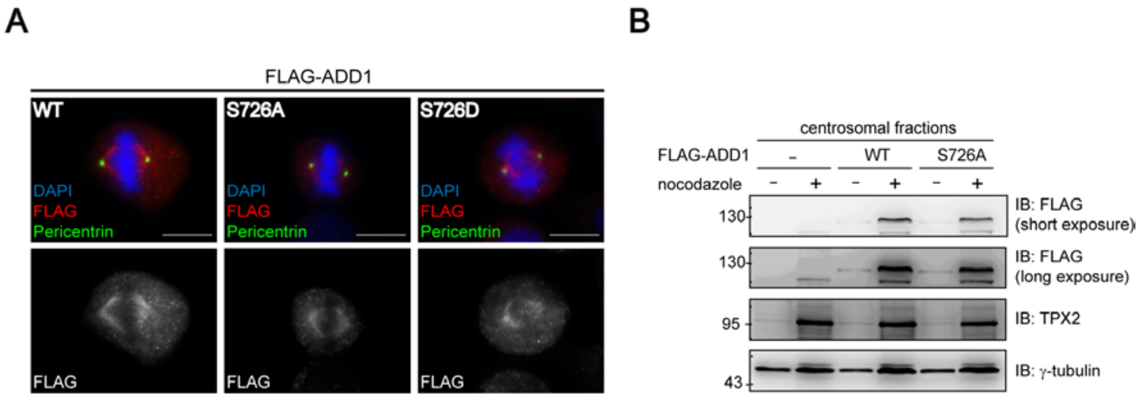

Appendix Figure S4. ADD1 S726 phosphorylation is not required for its centrosomal localization. (**A**) HeLa cells stably expressing FLAG-ADD1 WT, S726A, or S726D were stained for FLAG-ADD1, pericentrin, and DNA. Scale bars, 10 μm. (**B**) HeLa cells stably expressing FLAG-ADD1 WT or S726A remained asynchronized (-) or were synchronized at the M phase by nocodazole (+). The centrosomes were isolated from equal amounts of cells and analyzed by immunoblotting with the indicated antibodies.

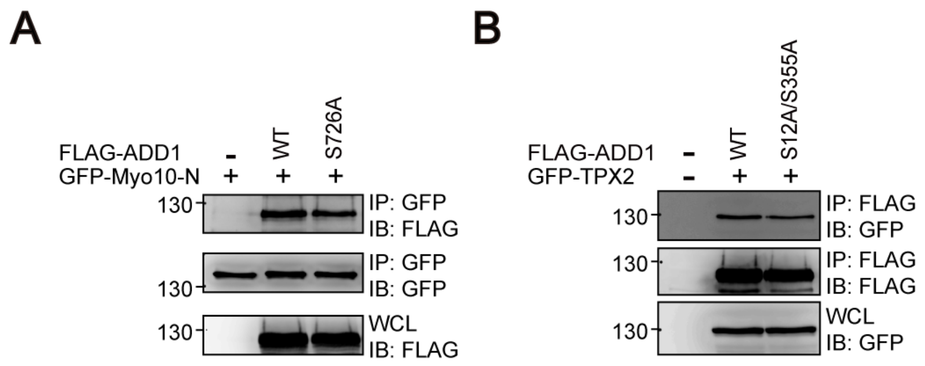

Appendix Figure S5. The interaction of ADD1 with Myo10 and TPX2 relies on serine phosphorylation at different sites. (**A**) GFP-fused N-terminus of Myo10 (GFP-Myo10-N) was transiently co-expressed with FLAG-ADD1 WT or S726A mutant in HEK293 cells. GFP-Myo10-N was immunoprecipitated (IP) by anti-GFP, and the immunocomplexes were analyzed by immunoblotting with anti-FLAG or anti-GFP. (**B**) GFP-TPX2 was co-expressed with FLAG-ADD1 WT or S12A/S355A mutant in HEK293 cells. Cell lysates were incubated with anti-FLAG (M2) affinity resins. The bound proteins were eluted from the resins with FLAG peptides and analyzed by immunoblotting (IB) with anti-FLAG or anti-GFP.
