# Supplementary material for: Adducin‐1 is essential for spindle pole integrity through its interaction with TPX2
Source: EMBO Rep. 2018 Jun 19;19(8):e45607. doi: 10.15252/embr.201745607 (PMC6073210; doi:10.15252/embr.201745607)
Supplement: Supplementary file 2 — Expanded View Figures PDF [file EMBR-19-e45607-s002.pdf]

Expanded View Figures

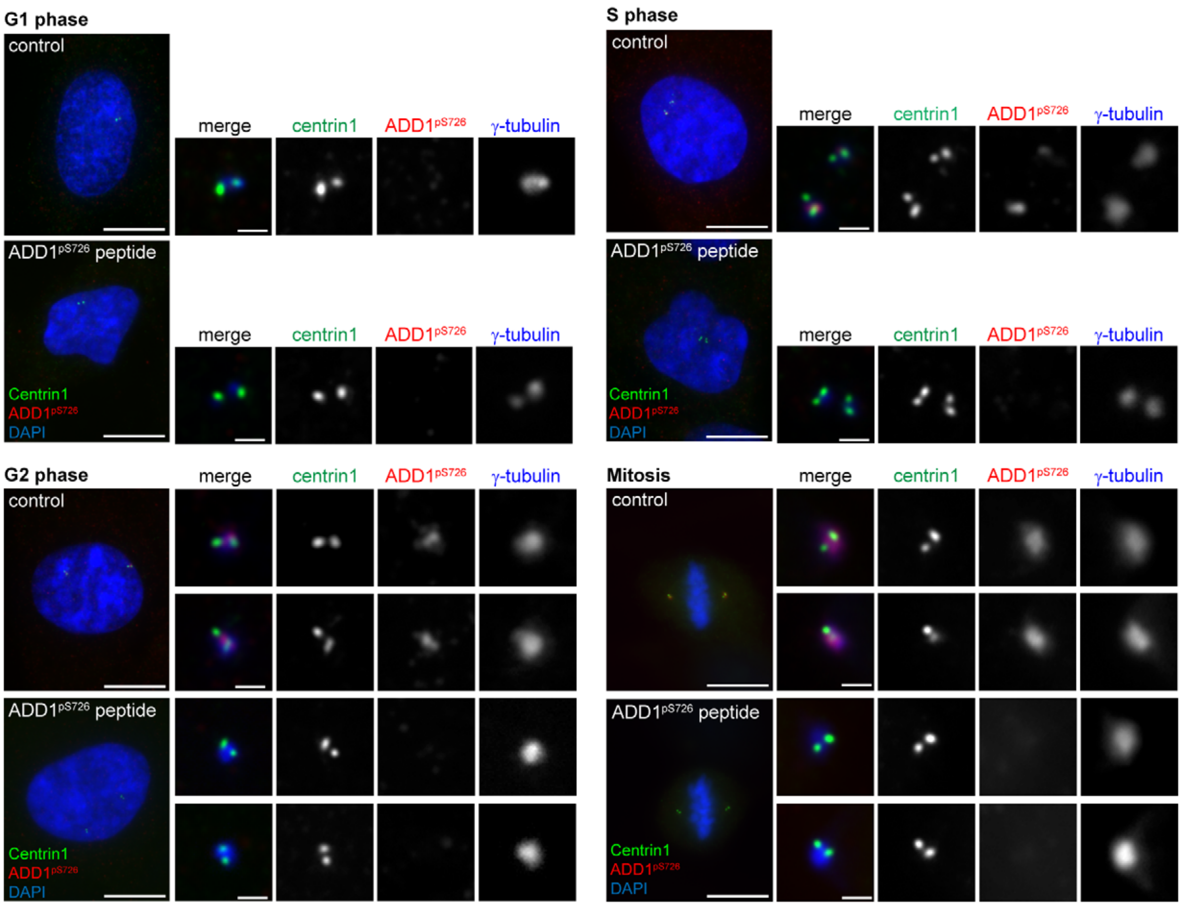

Figure EV1. Verification of the specificity of the anti-ADD1 pS726 antibody in immunofluorescence staining.
RPE1 cells at the indicated cell cycle stages were stained for centrin1 (green), ADD1 pS726 (red), and γ-tubulin (blue). For phosphopeptide competition, the anti-ADD1 pS726 antibody was pre-mixed with a phosphopeptide (ADD1 pS726 peptide) before staining. DNA was visualized by DAPI staining. High magnification images of each centrosome are shown on the right. Scale bars, 10 μm (main image) and 1 μm (zoomed images).

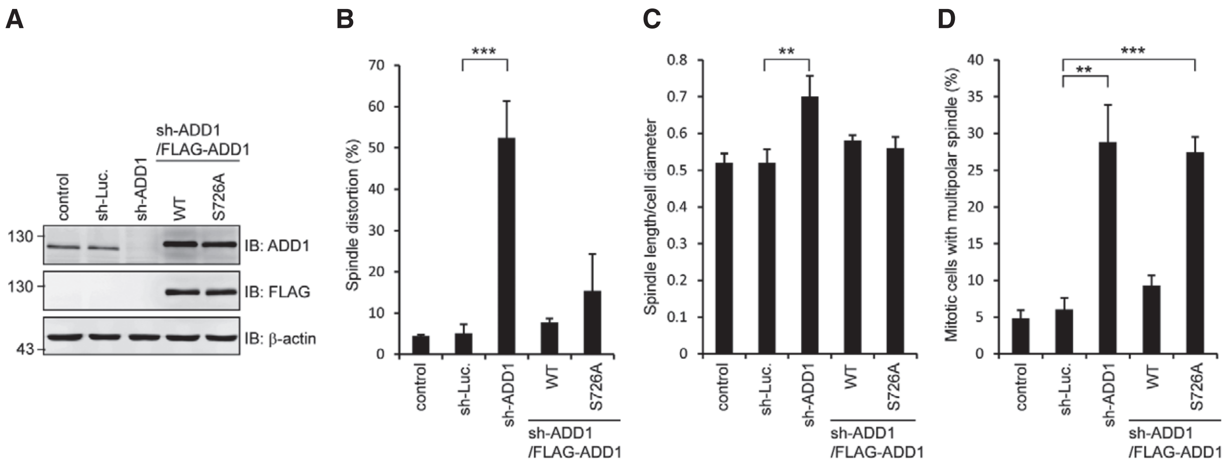

Figure EV2.

 © 2018 The Authors

Figure EV2. FLAG-ADD1 S726A restores the defects of spindle distortion and elongation, but not multipolar spindle formation, induced by ADD1 depletion.

A HeLa cells were infected with lentiviruses expressing shRNAs to ADD1 (sh-ADD1) or luciferase (sh-Luc.) as a control. FLAG-ADD1 WT or the S726A mutant was re-expressed in the cells whose endogenous ADD1 had been depleted. Equal amounts of whole-cell lysates were analyzed by immunoblotting (IB) with the indicated antibodies.

B The percentage of distorted spindles in the total number of mitotic cells was measured (367–408 mitotic cells were counted in each group).

C The ratio of spindle length to cell diameter was measured (80–112 mitotic cells were counted in each group).

D The percentage of multipolar spindles in the total number of mitotic cells was measured (405–509 mitotic cells were counted in each group).

Data information: In (B–D), values (mean ± s.d.) are from three independent experiments. Statistical significance of differences is assessed with a Student's *t*-test: for (B), ***P = 0.0009; for (C), **P = 0.0092; for (D), **P = 0.0018, ***P = 0.0002.
Source data are available online for this figure.

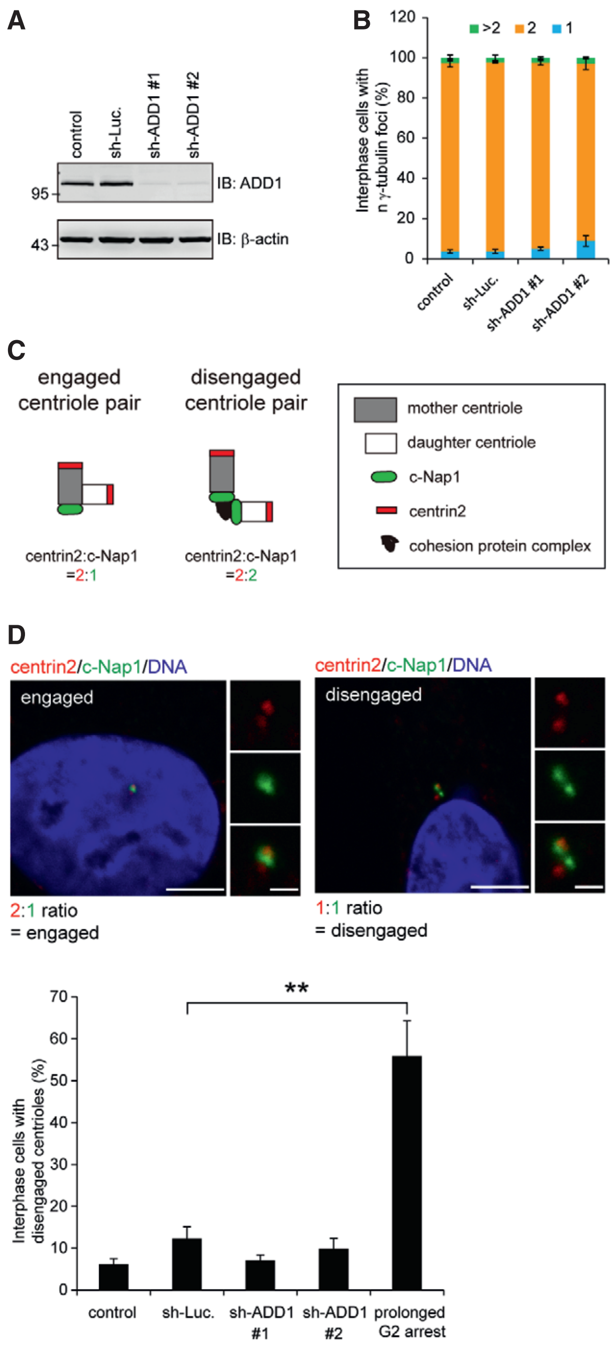

Figure EV3. Increased number of centrosome foci during mitosis in ADD1-depleted cells is not caused by centrosome overduplication or centriole disengagement at interphase.

A HeLa cells were infected with lentiviruses expressing shRNAs to ADD1 (sh-ADD1 #1 and sh-ADD1 #2) or luciferase (sh-Luc.) as a control. The expression levels of ADD1 and β-actin (loading control) were analyzed by immunoblotting with the indicated antibodies.

B The percentage of interphase cells with 1, 2, or > 2 γ-tubulin foci was assessed (1,085–1,311 cells were counted in each group).

C Schematic representation of the engaged and disengaged centriole pairs. Engaged centriole pairs have one C-Nap1 focus and two centrin2 foci, whereas disengaged centriole pairs have both foci of C-Nap1 and centrin2.

D HeLa cells were infected with lentiviruses expressing shRNAs to ADD1 (sh-ADD1 #1 and sh-ADD1 #2) or luciferase (sh-Luc.) as a control. In the control experiment, the cells were treated with a CDK1 inhibitor, RO-3306, for 16 h to induce arrest at the G2 phase. The cells were stained for centrin2 (red), c-Nap1 (green), and DNA (blue). Quantification of the interphase cells with disengaged centrioles (700–1,690 cells were counted in each group). Scale bars, 5 μm (main image) and 1 μm (zoomed images).

Data information: In (B and D), values (mean ± s.d.) are from three independent experiments. **P = 0.001 (Student's *t*-test).
Source data are available online for this figure.

© 2018 The Authors

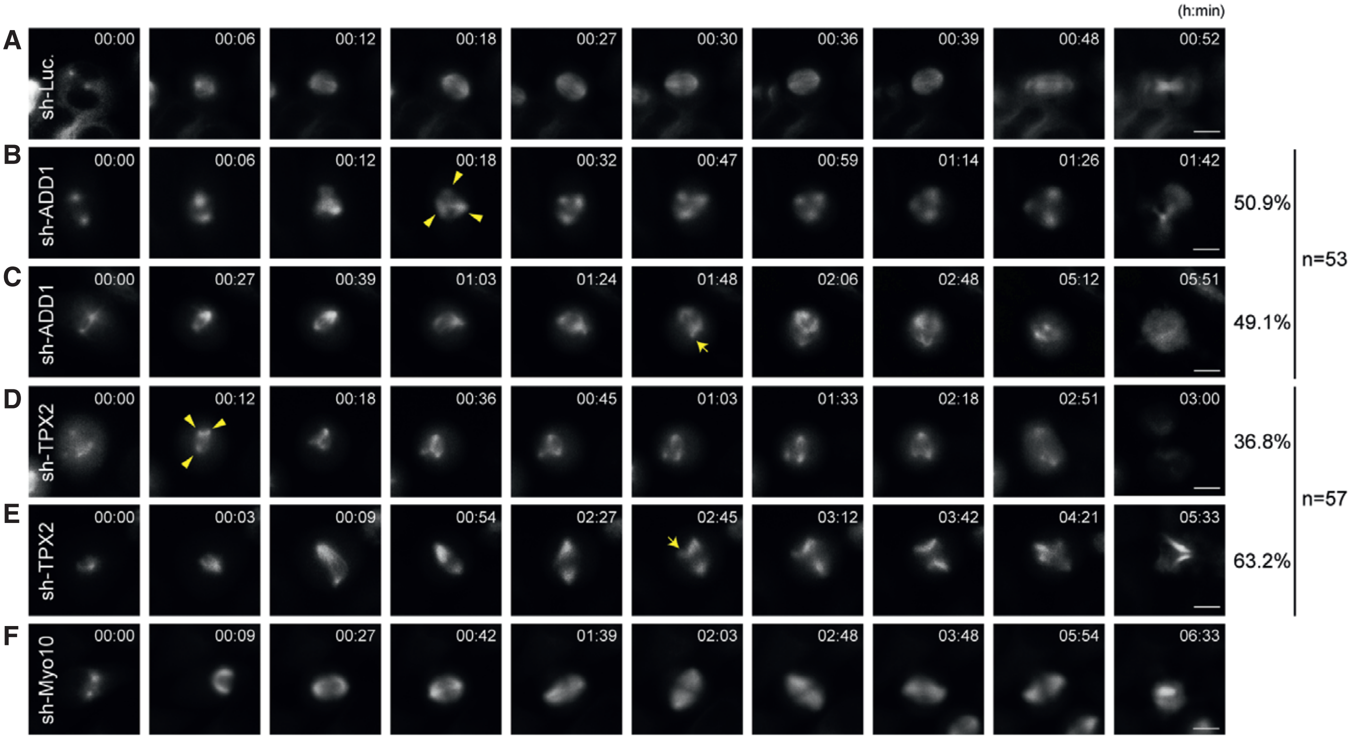

Figure EV4. Mitotic arrest contributes to, but does not fully explain ADD1 depletion-induced spindle multipolarity.

A–F HeLa cells stably expressing mCherry-tubulin were infected with lentiviruses expressing shRNAs to (A) luciferase (sh-Luc.) as a control, (B, C) ADD1 (sh-ADD1), (D, E) TPX2 (sh-TPX2), or (F) Myo10 (sh-Myo10). The cells were monitored with time-lapse microscopy. Images were captured every 3 min for 18 h. Arrowheads indicate the multiple spindle poles occurs within 1 h upon nuclear envelope breakdown. Arrows indicate the multiple spindle poles occurs more than 1 h after entering mitosis. Scale bars, 10 μm. The percentages of these two categories in the total counted samples (53 for sh-ADD1 and 57 for sh-TPX2) are indicated to the right of the micrographs.

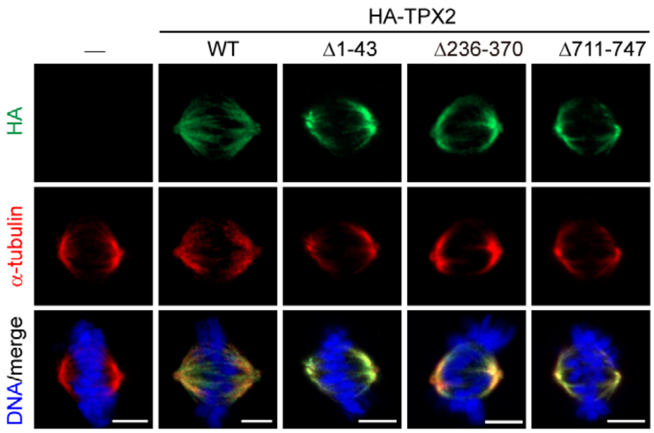

Figure EV5. TPX2 deletion mutants retain their capability to associate with mitotic spindles.
HeLa cells stably expressing HA-TPX2 WT and the mutants were stained for HA-TPX2, α-tubulin, and DNA. Scale bars, 5 μm.

© 2018 The Authors
