# Supplementary figures and images for: Adducin‐1 is essential for spindle pole integrity through its interaction with TPX2
Source: EMBO Rep. 2018 Jun 19;19(8):e45607. doi: 10.15252/embr.201745607 (PMC6073210; doi:10.15252/embr.201745607)

Source data for Appendix Figure S3

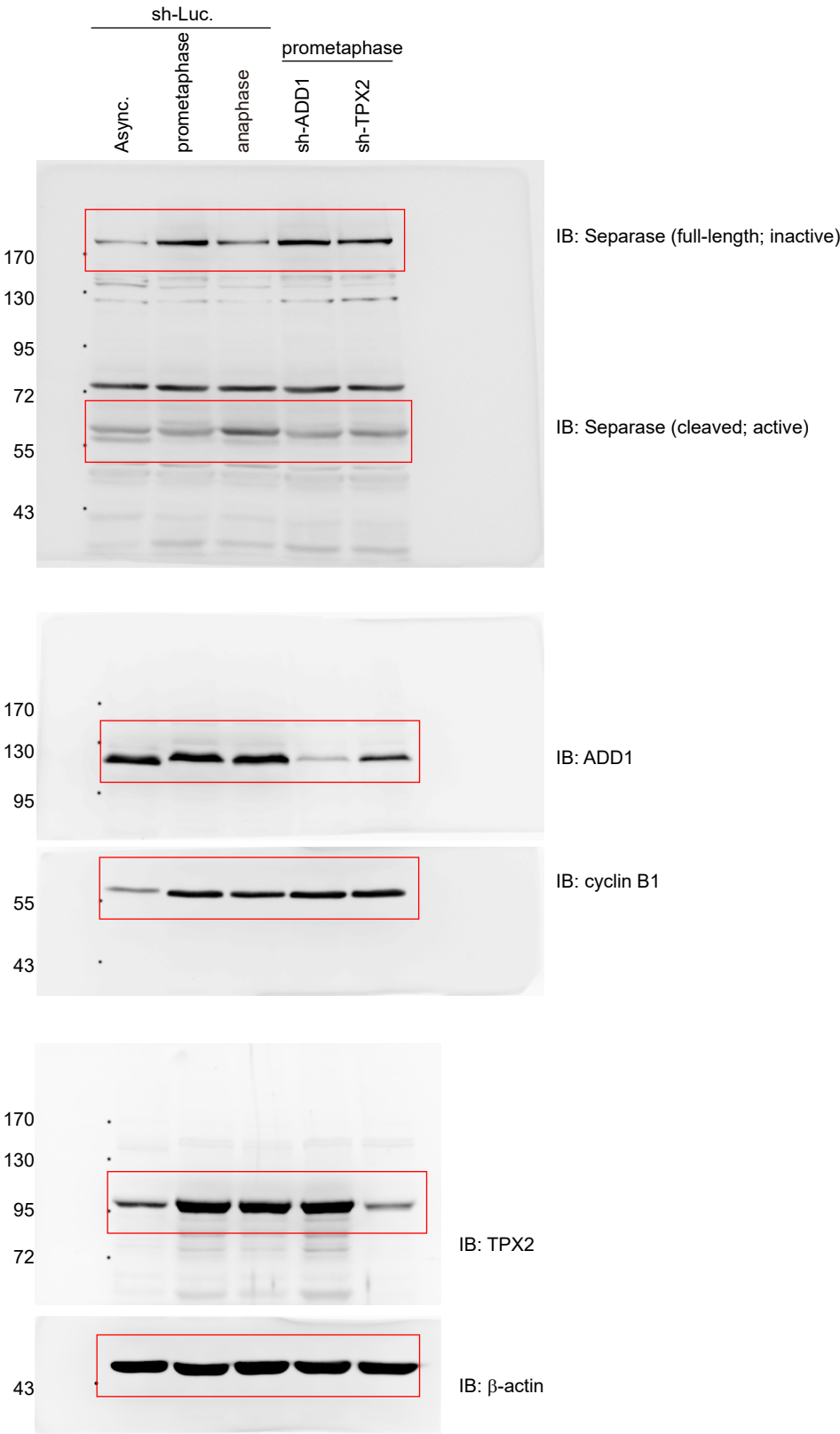

Supplement: Supplementary file 3 — Source Data for Expanded View and Appendix [file EMBR-19-e45607-s007.zip › EMBOR-2017-45607-T_SourceDataForAppendixFigS3.pdf]

B

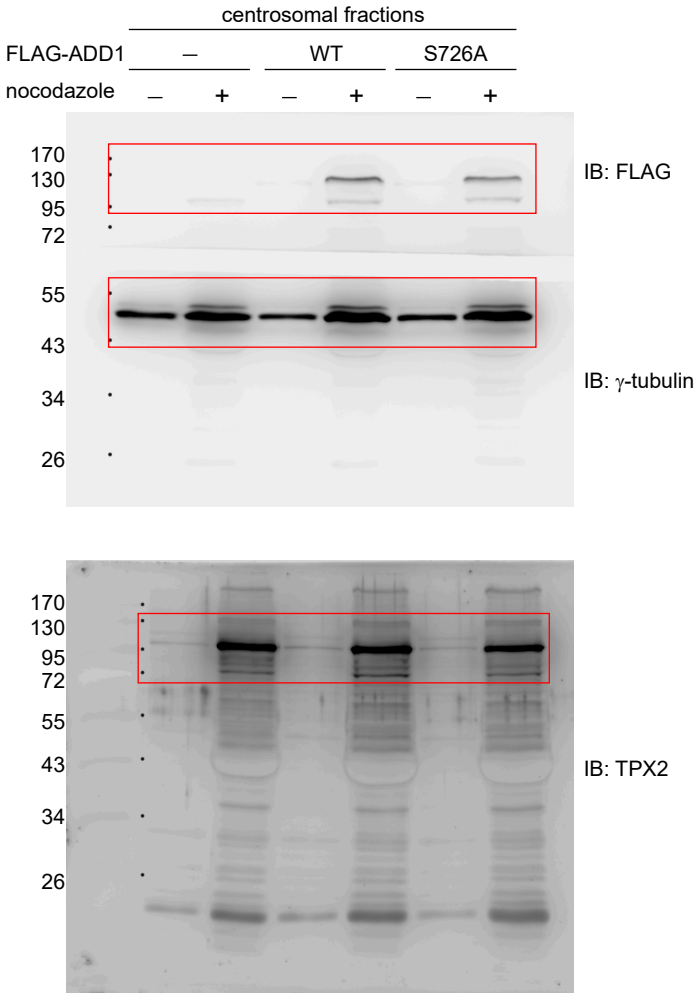

Supplement: Supplementary file 3 — Source Data for Expanded View and Appendix [file EMBR-19-e45607-s007.zip › EMBOR-2017-45607-T_SourceDataForAppendixFigS4.pdf]

Source data for Appendix Figure S5

A

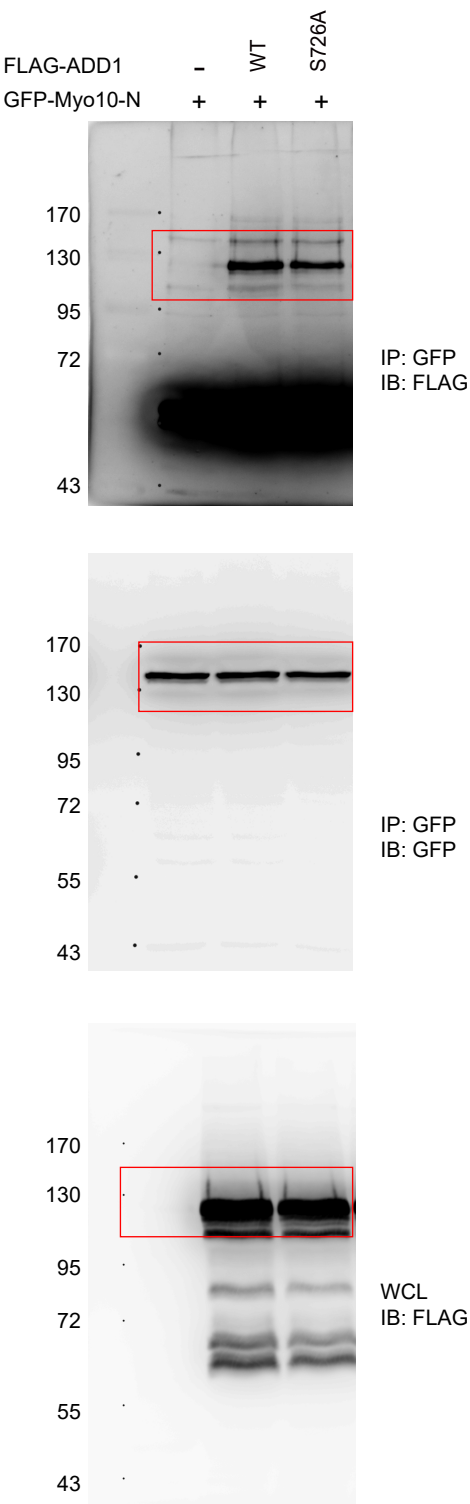

B

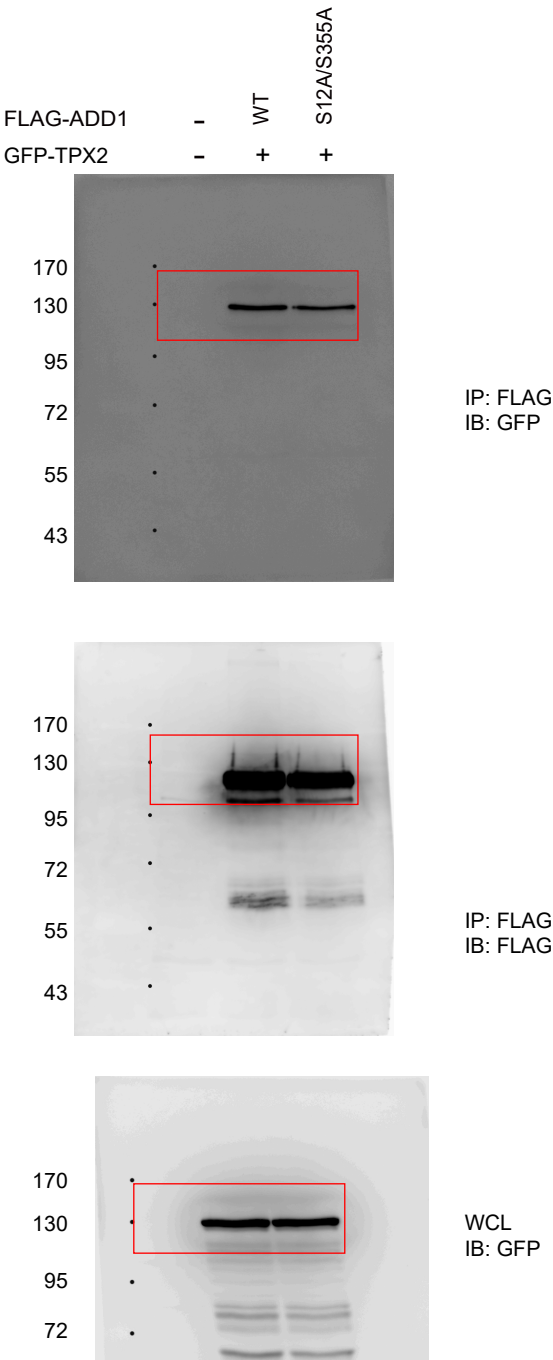

Supplement: Supplementary file 3 — Source Data for Expanded View and Appendix [file EMBR-19-e45607-s007.zip › EMBOR-2017-45607-T_SourceDataForAppendixFigS5.pdf]

A

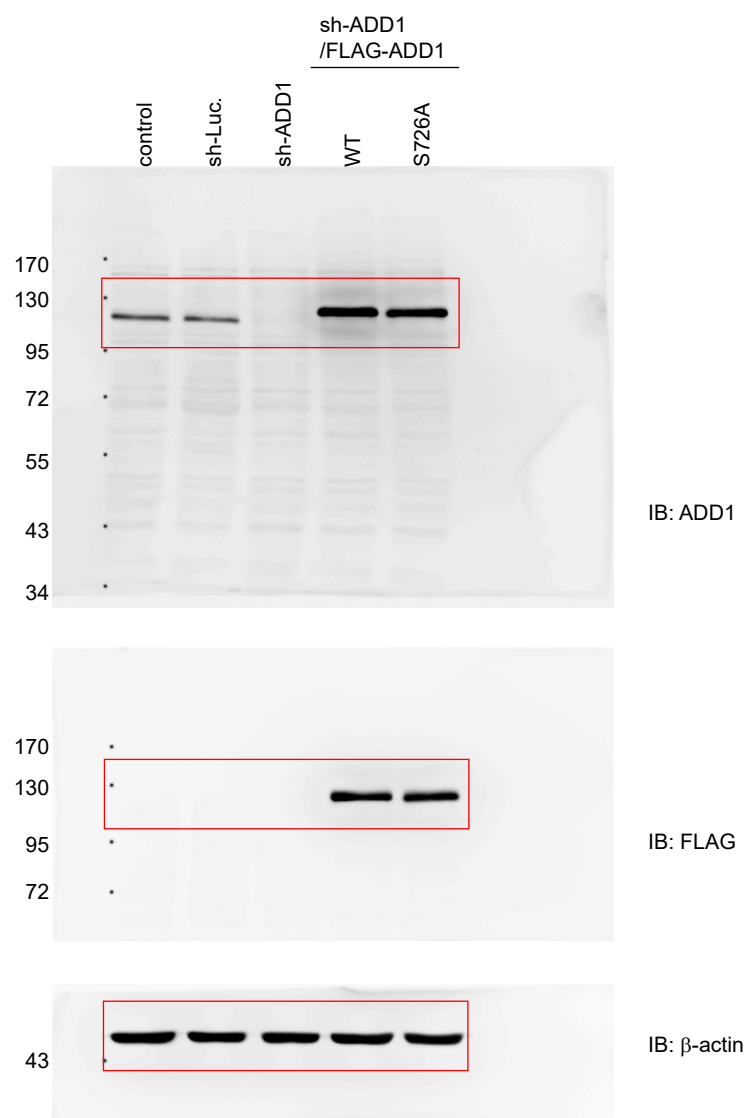

Supplement: Supplementary file 3 — Source Data for Expanded View and Appendix [file EMBR-19-e45607-s007.zip › EMBOR-2017-45607-T_SourceDataForFigureEV2.pdf]

A

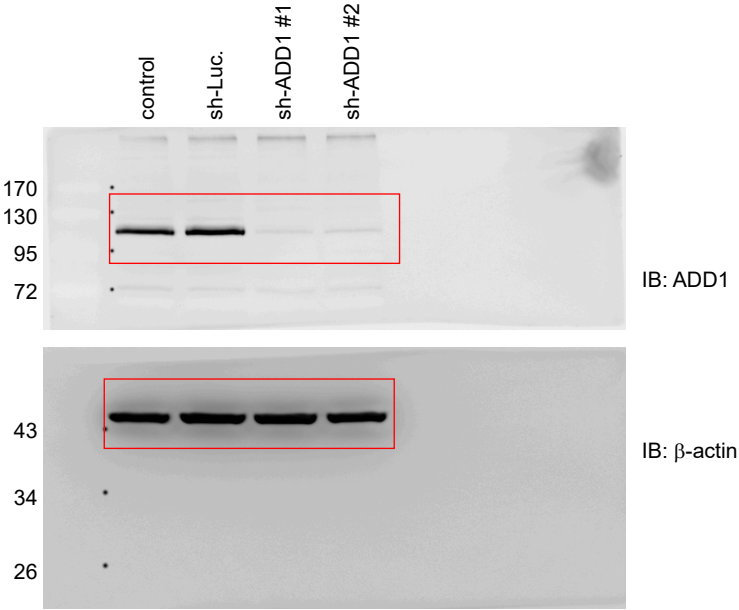

Supplement: Supplementary file 3 — Source Data for Expanded View and Appendix [file EMBR-19-e45607-s007.zip › EMBOR-2017-45607-T_SourceDataForFigureEV3.pdf]

A

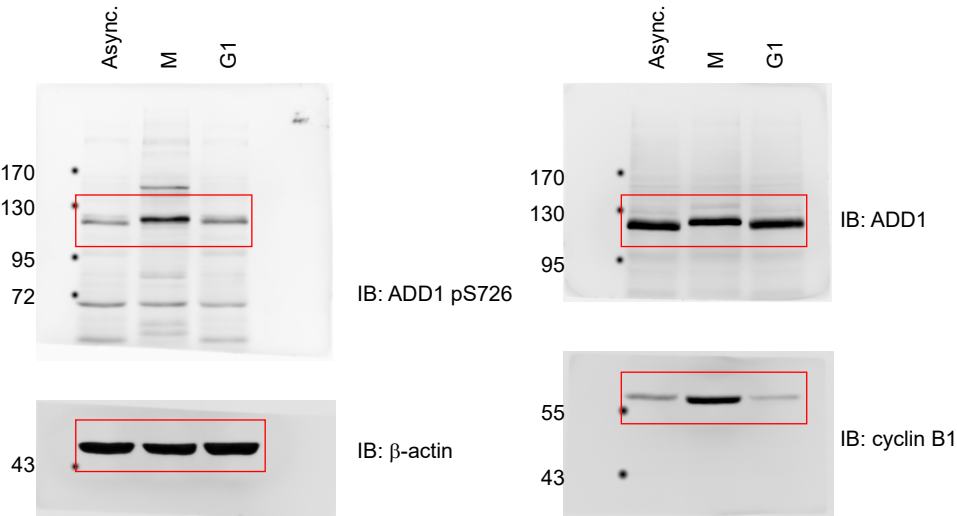

Supplement: Supplementary file 5 — Source Data for Figure 1 [file EMBR-19-e45607-s003.pdf]

A

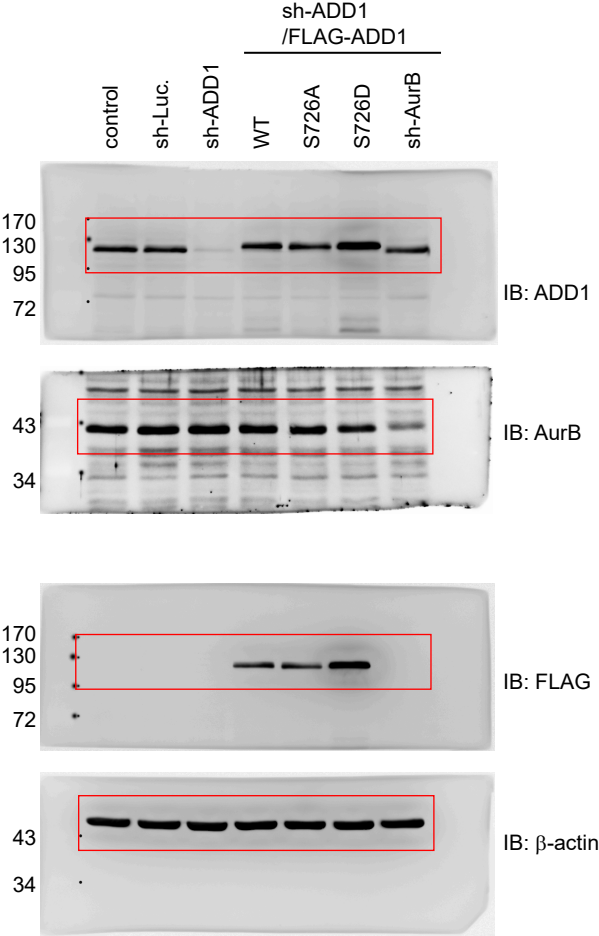

Supplement: Supplementary file 6 — Source Data for Figure 3 [file EMBR-19-e45607-s004.pdf]

Source data for Figure 5

A

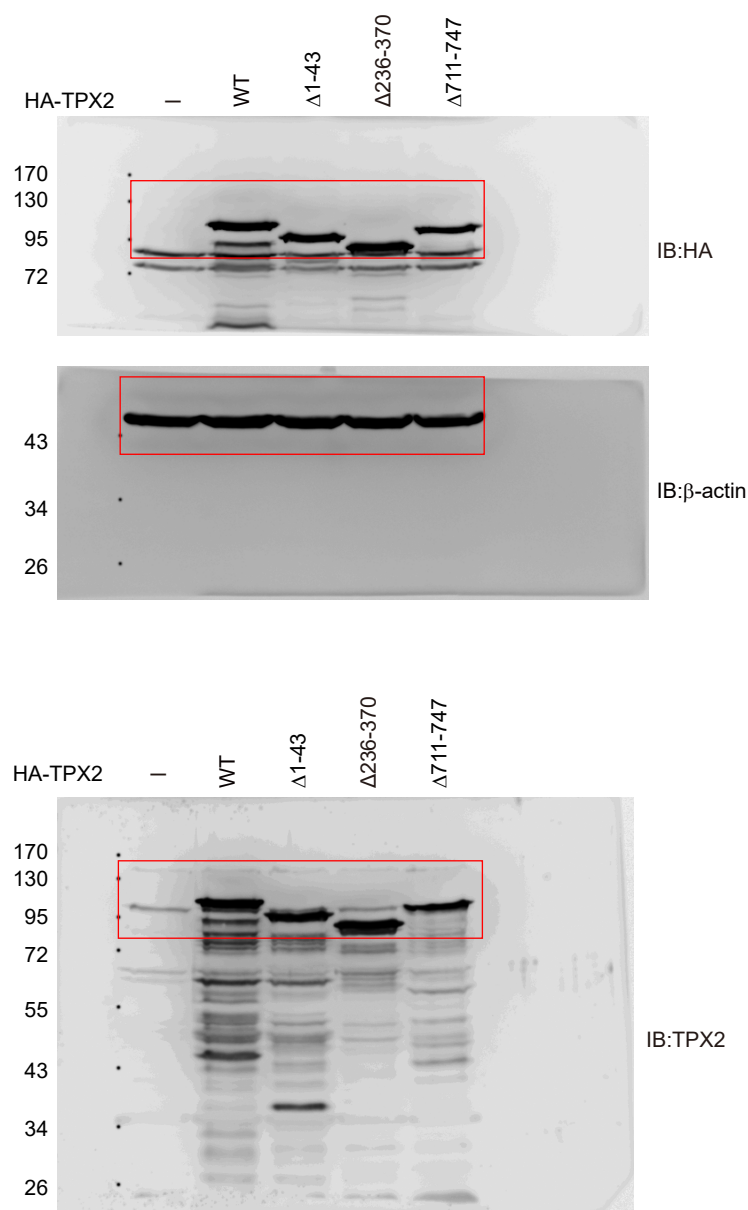

B

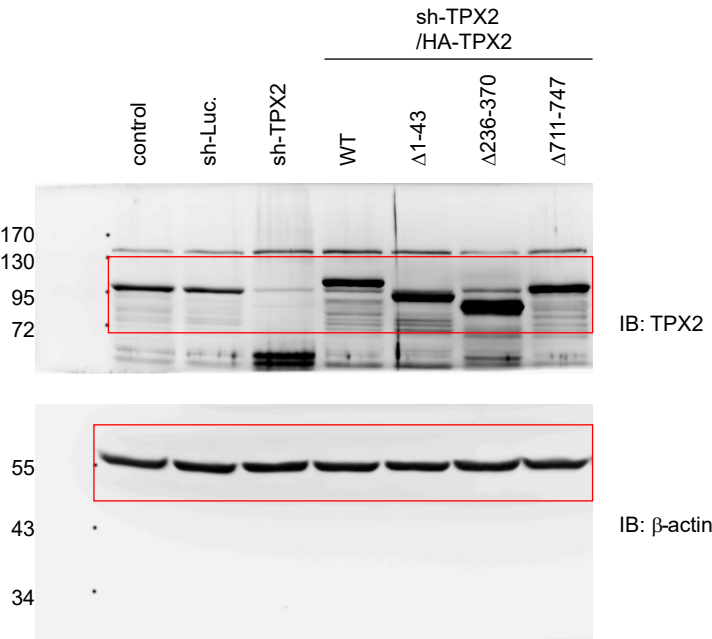

C

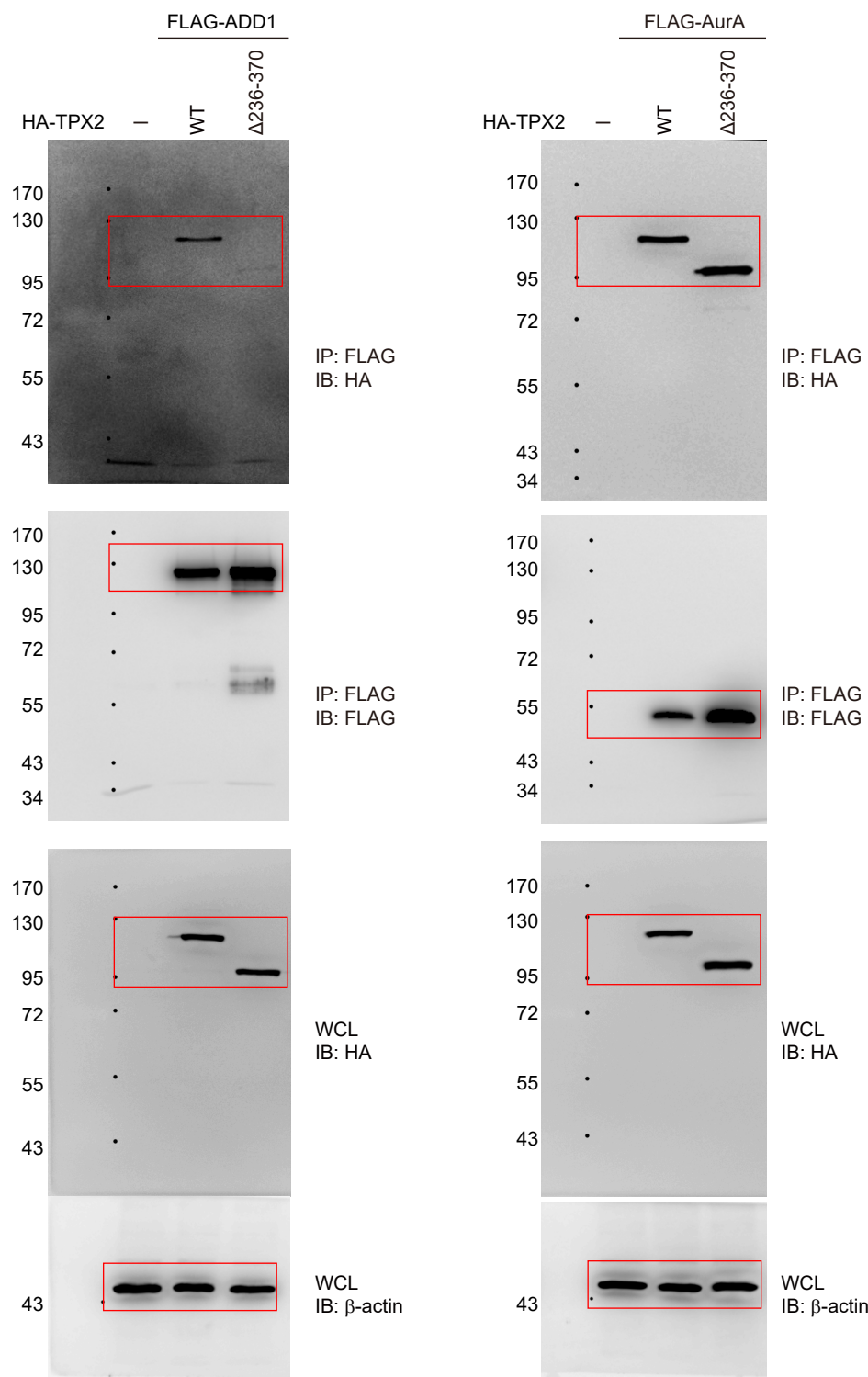

Supplement: Supplementary file 8 — Source Data for Figure 5 [file EMBR-19-e45607-s006.pdf]
