# Supplementary material for: Adducin‐1 is essential for spindle pole integrity through its interaction with TPX2
Source: EMBO Rep. 2018 Jun 19;19(8):e45607. doi: 10.15252/embr.201745607 (PMC6073210; doi:10.15252/embr.201745607)
Supplement: Supplementary file 7 — Source Data for Figure 4 [file EMBR-19-e45607-s005.pdf]

Source data for Figure 4

A

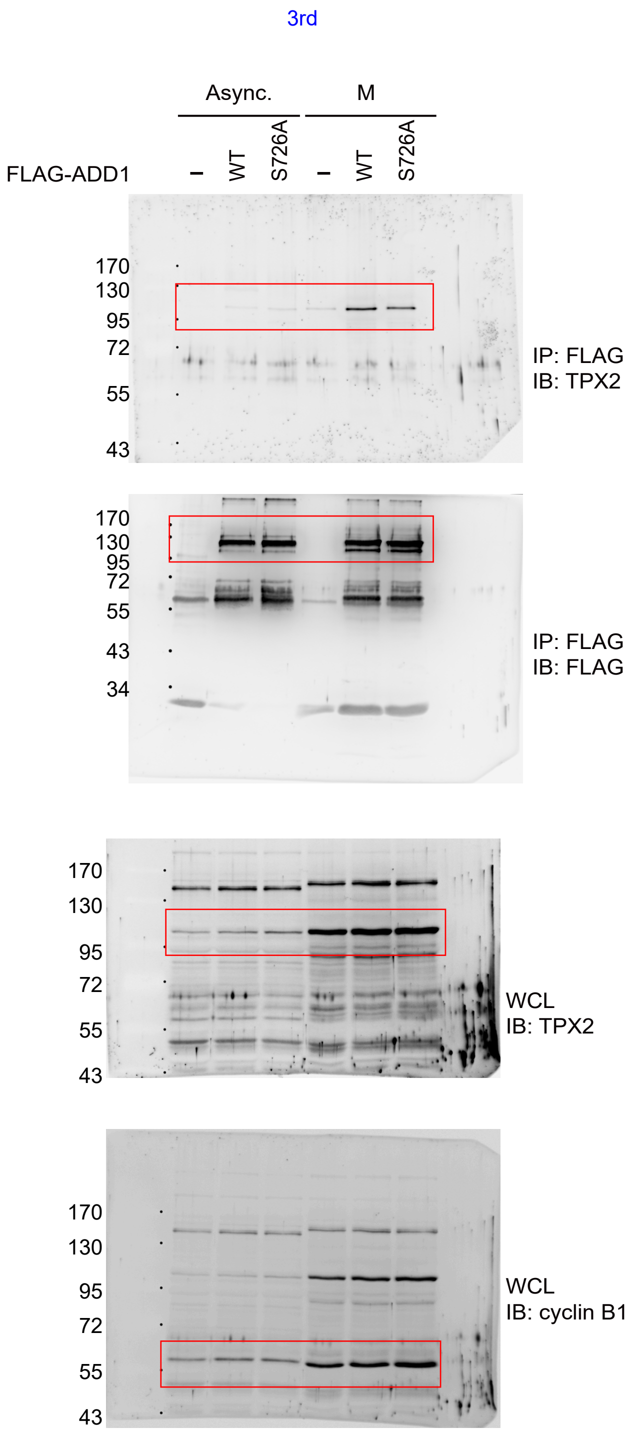

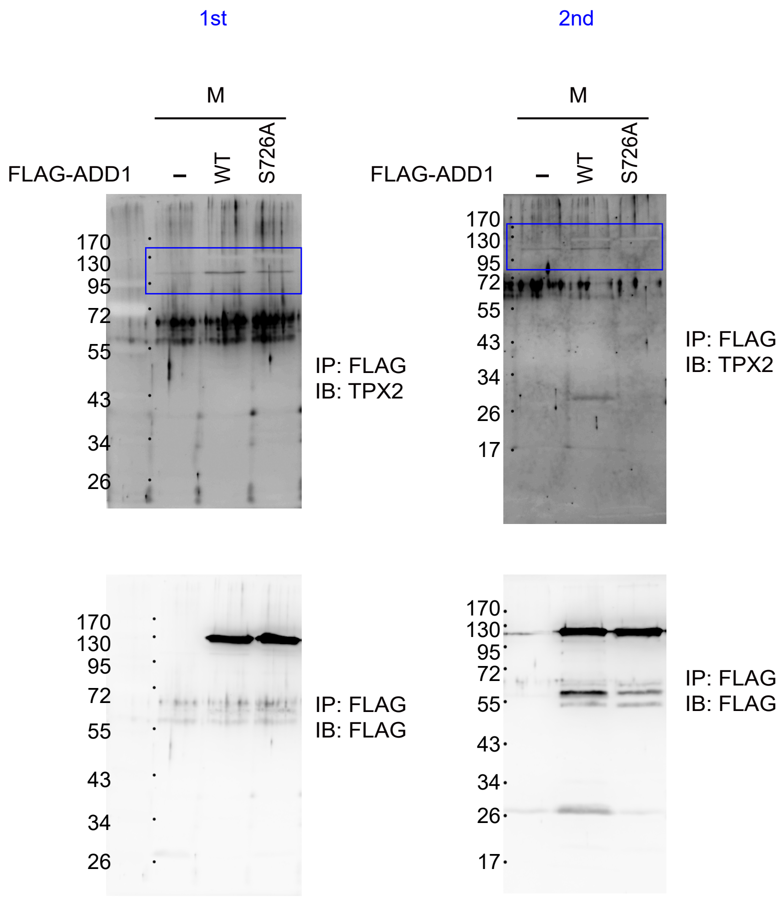

	WT	S726A
1st	1	0.491456
2nd	1	0.150341
3rd	1	0.559967
mean	1	0.400588
s.d.	0	0.219411
P-value		0.0091

Source data for Figure 4

B

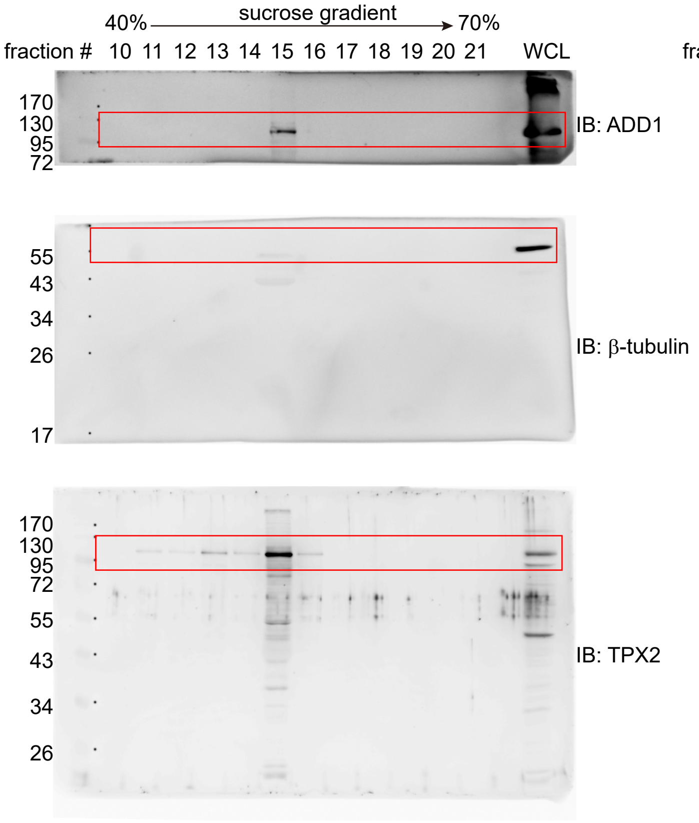

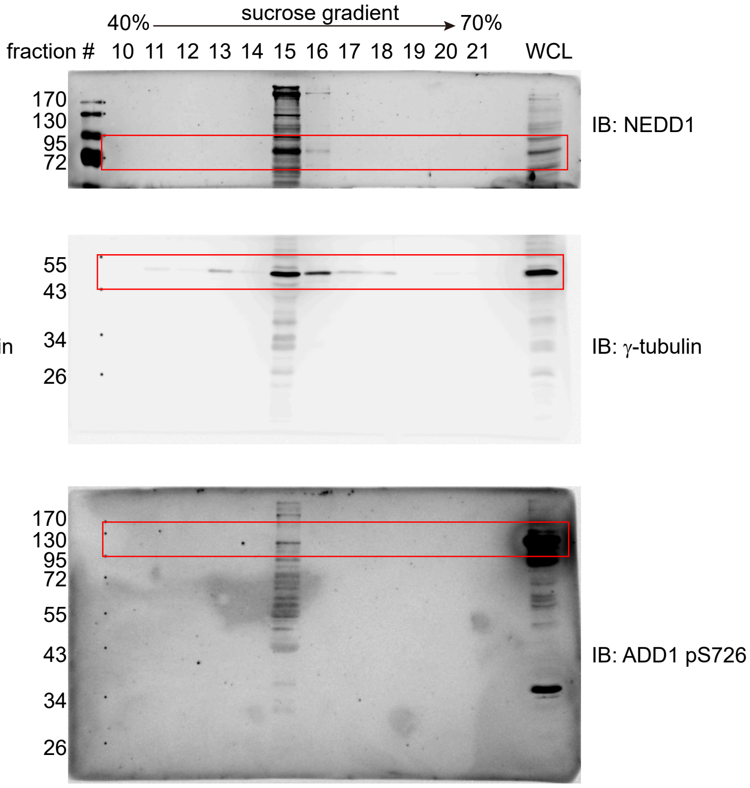

Source data for Figure 4

E

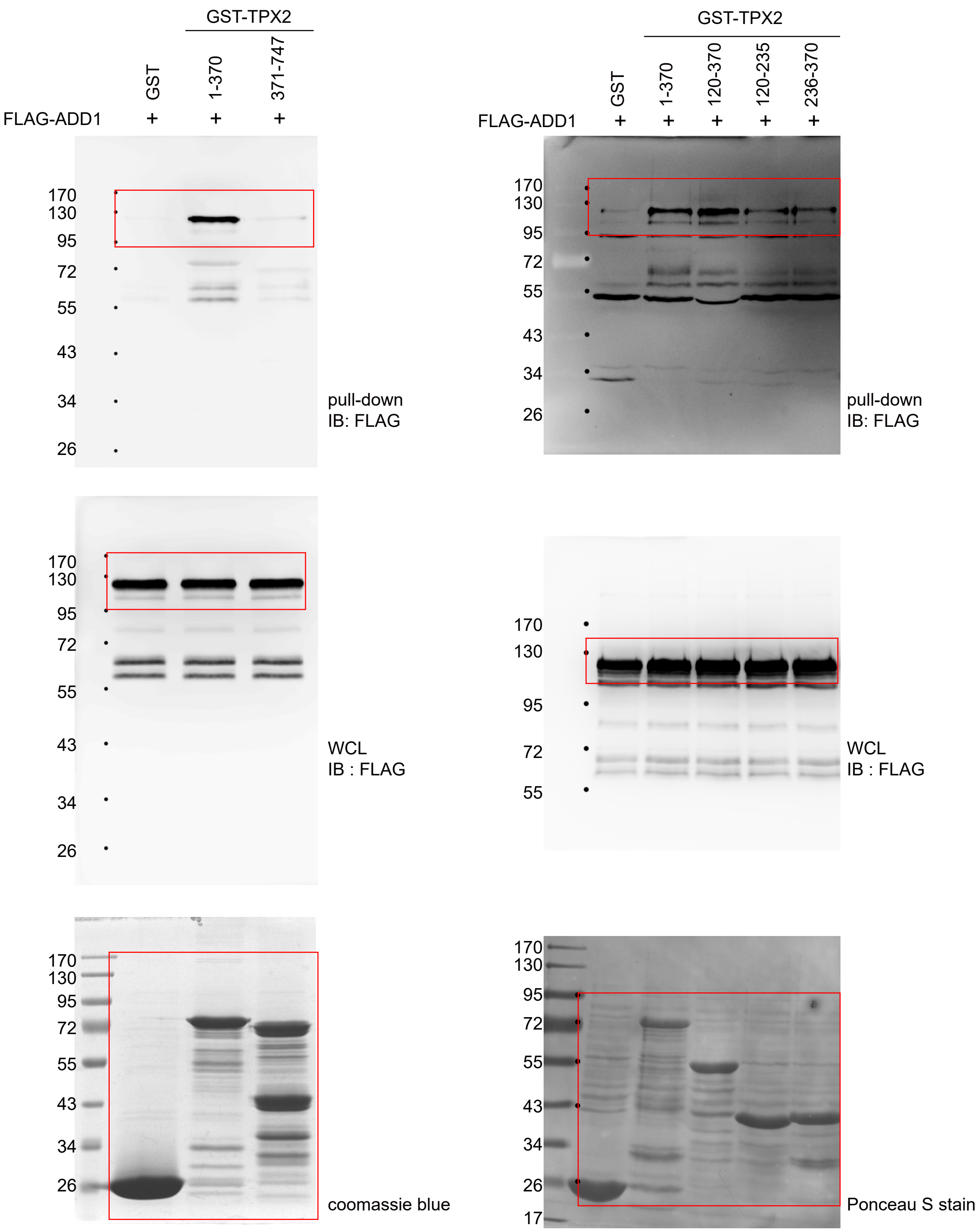

Source data for Figure 4

F

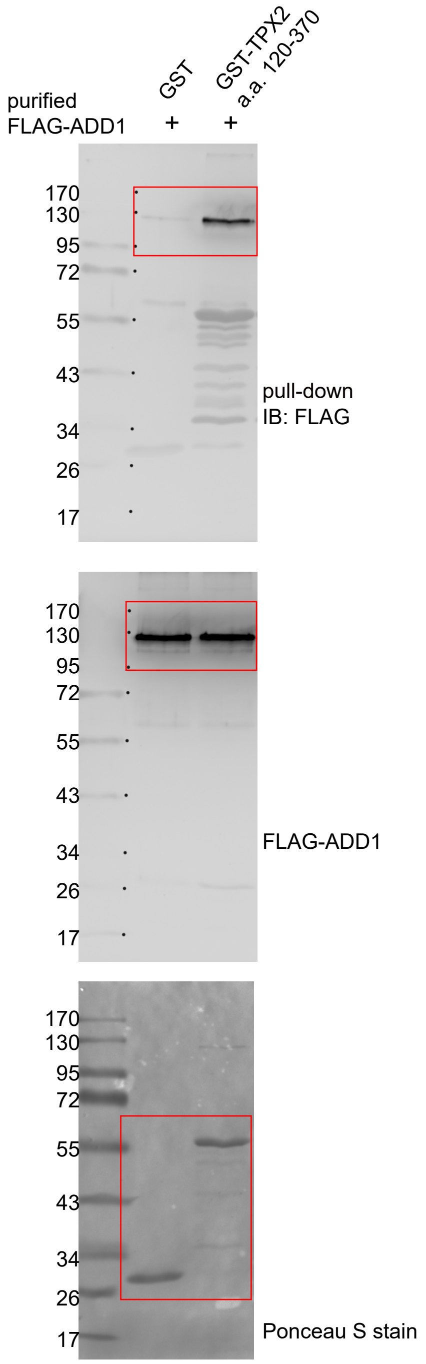

G

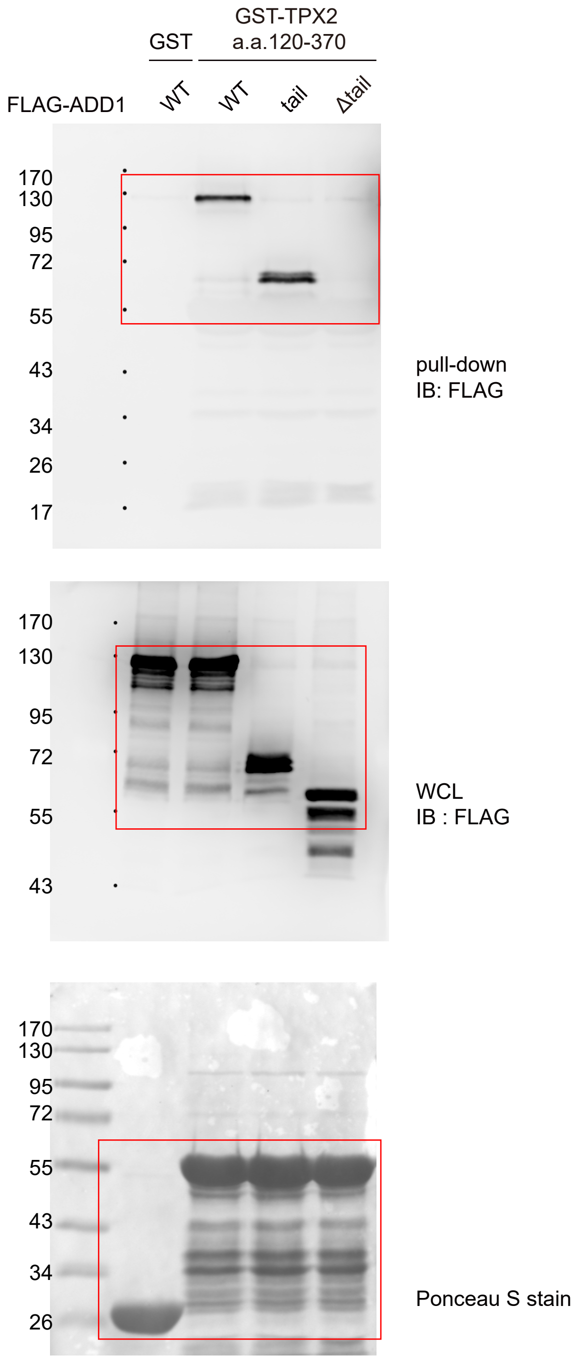

Source data for Figure 4

H

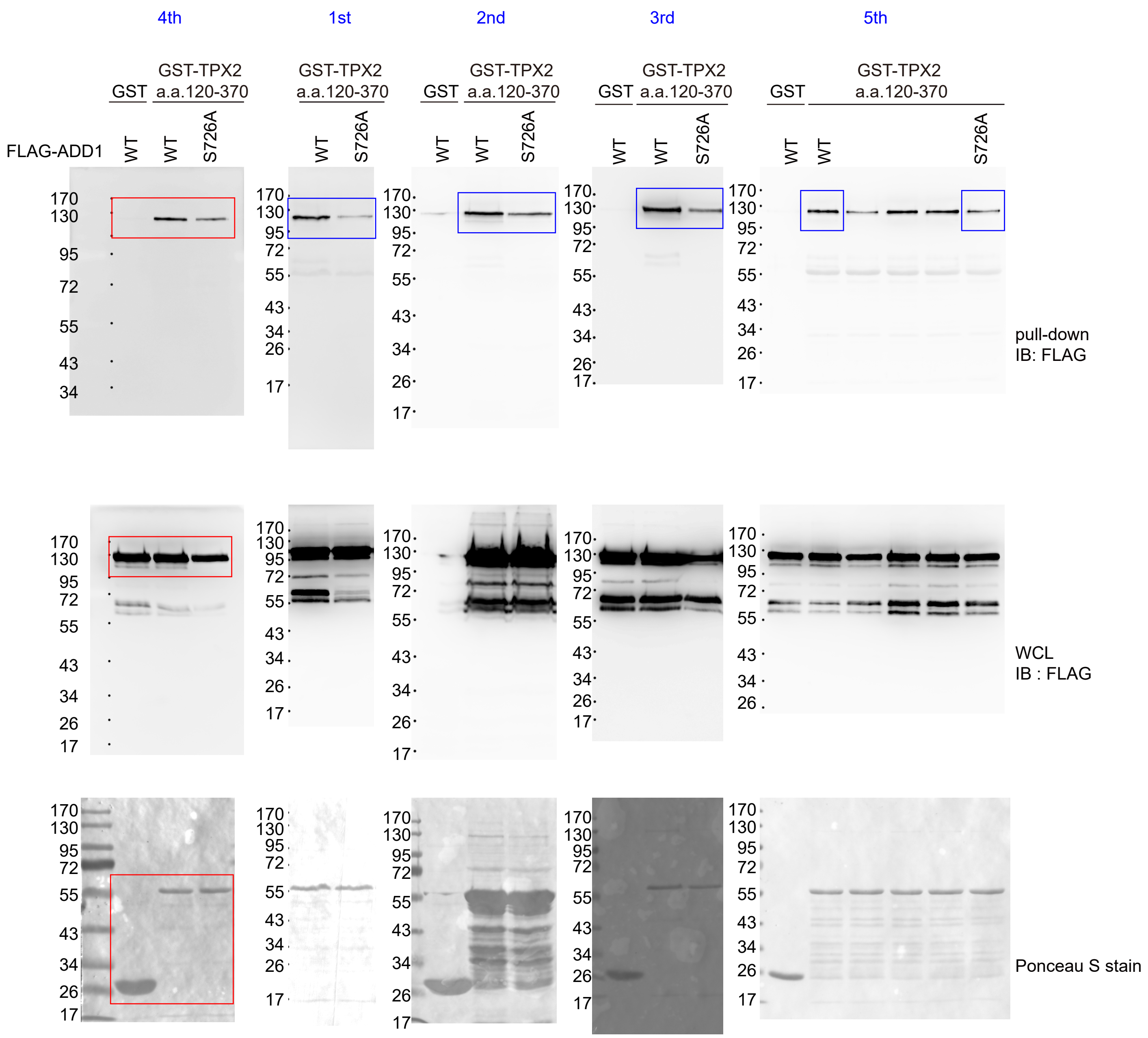

	WT	S726A
1st	1	0.155227
2nd	1	0.600589
3rd	1	0.304897
4th	1	0.431667
5th	1	0.517144
mean	1	0.401905
s.d.	0	0.175998
P-value		6.00E-05
